# Self-Locking Optoelectronic Tweezers for Single-Cell and Microparticle Manipulation across a Large Area in High Conductivity Media

**DOI:** 10.1038/srep22630

**Published:** 2016-03-04

**Authors:** Yajia Yang, Yufei Mao, Kyeong-Sik Shin, Chi On Chui, Pei-Yu Chiou

**Affiliations:** 1 Mechanical and Aerospace Engineering Department, University of California, Los Angeles, USA; 2 Electrical Engineering Department, University of California, Los Angeles, USA; 3 Bioengineering Department, University of California, Los Angeles, USA

## Abstract

Optoelectronic tweezers (OET) has advanced within the past decade to become a promising tool for cell and microparticle manipulation. Its incompatibility with high conductivity media and limited throughput remain two major technical challenges. Here a novel manipulation concept and corresponding platform called Self-Locking Optoelectronic Tweezers (SLOT) are proposed and demonstrated to tackle these challenges concurrently. The SLOT platform comprises a periodic array of optically tunable phototransistor traps above which randomly dispersed single cells and microparticles are self-aligned to and retained without light illumination. Light beam illumination on a phototransistor turns off the trap and releases the trapped cell, which is then transported downstream via a background flow. The cell trapping and releasing functions in SLOT are decoupled, which is a unique feature that enables SLOT’s stepper-mode function to overcome the small field-of-view issue that all prior OET technologies encountered in manipulation with single-cell resolution across a large area. Massively parallel trapping of more than 100,000 microparticles has been demonstrated in high conductivity media. Even larger scale trapping and manipulation can be achieved by linearly scaling up the number of phototransistors and device area. Cells after manipulation on the SLOT platform maintain high cell viability and normal multi-day divisibility.

Optoelectronic Tweezers (OET)^1^, also known as optically induced dielectrophoresis (ODEP), has emerged as a useful tool for manipulation of cells and microparticles in a variety of biomedical applications. It has been shown to be able to trap and manipulate semiconductor and metallic nanowires^2^, micro- and nano-beads^3^,^4^,^5^,^6^,^7^,^8^, DNA molecules^9^, droplets^10^, and various types of biological cells^11^,^12^,^13^,^14^,^15^,^16^,^17^. Photoconductive materials including hydrogenated amorphous silicon (a-Si:H)^1^, single crystalline silicon^18^, P3HT:PCBM^19^, titanium oxide phthalocyanine^20^ and lithium niobate^21^ have been utilized to create light-patterned virtual electrodes. Integration of OET with microfluidic devices for adding extra functions such as media swapping^22^ and single-cell analysis^23^,^24^ has further expanded the utilities of OET technologies.

However, most conventional OET devices cannot operate in high conductivity media, including regular physiological buffers, due to the fact that only limited photocurrents can be generated in the aforementioned photoconductive materials. The only exception is the single crystalline silicon phototransistor-based OET^18^,^25^ which allows optical manipulation of single cells in regular physiological buffers without pre-suspending biological cells in low ionic strength buffers that affect cells’ regular physiological functions and viability^26^.

In addition to the concern of medium conductivity, conventional OET devices only support the operation across a small field-of-view (FOV) to maintain the optical resolution required for single-cell manipulation. Up to date, the largest single-cell manipulation area has been demonstrated by holographic OET^27^ by utilizing lens-free imaging techniques to overcome the small FOV issue. Yet, the issues of single-cell manipulation over a large area are not completely solved. The remaining challenge comes from the resolution of the light beams required for patterning virtual electrodes on OET. Such light patterns are usually generated through a spatial light modulator such as a digital micromirror device (DMD) or a scanning mirror. Under either circumstance, the manipulation area is limited by the FOV of the objective lenses used to project the beams. Since a lens with a large FOV typically comes with a low numerical aperture (NA), it cannot provide the light beam a resolution required for single-cell manipulation.

Furthermore, a blurry light beam also causes a significant decrease in DEP forces, which are linearly proportional to the gradient of electric field strength (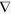
E^2^). In general, OET operation relies on sharp virtual electrode edges to create DEP forces for small particle manipulation. When a blurry virtual electrode is created, the transition distance between the ON and OFF regions is long. Provided the constraint that the maximum electrical voltage one can apply to drive an OET platform is fixed, a long ON to OFF transition distance means a low electric field gradient and a small DEP force. For example, if a 10x  objective lens is required to manipulate single cells across a 1 mm^2^ area, the DEP forces on cells will decrease by 10 times if the light pattern is enlarged to 1 cm^2^ and fail to actuate cells, even when there is sufficient light power to fully turn on virtual electrodes.

In summary, single-cell manipulation on OET-based platforms over a large area in high conductivity media is limited by several fundamental barriers. Prior works on phototransistor-based OET and holographic OET are only partial solutions. In this paper, we present a novel manipulation concept and corresponding platform called SLOT that constitutes a practical and feasible solution to overcome these barriers.

**Design and Operation Principle of SLOT**. SLOT is realized by laying out a high-density array of ring-shaped, phototransistor controlled electrodes for single-cell trapping (Fig. 1). The size of these electrodes is adjusted to trap only one cell. Operation of SLOT needs a background fluid flow for introducing cells and microparticles as well as providing fluid viscous forces on trapped cells (Fig. 1(b)). When an AC voltage is applied to the top and the bottom electrodes on a SLOT platform, single cells or microparticles will be immediately locked by nearby ring-shaped phototransistors across the active area without light illumination (see Supplementary Video 1). This locking function is achieved by properly choosing an appropriate AC frequency and a thin, high k dielectric layer on the bottom electrode to allow partial voltage drop in the liquid layer without light illumination (see Supplementary Fig. 1). Since the phototransistors are not illuminated, the center electrodes they control are still off in the dark state. Electric field strength on top of these electrodes is weaker than other regions on the chip, which generates negative DEP forces on neighboring cells and locks them into these traps. For cells or particles not locked, the background flow brings them to the downstream until they are locked by empty phototransistors. When a light beam illuminates a phototransistor, the photocurrent increases to turn on its center electrode to create a stronger electric field than the background, which pushes the locked cell out of the illuminated trap. The released cell is carried to the downstream by the background flow.

SLOT introduces a new manipulation concept different from what was commonly applied in conventional OET systems, in which DEP forces are generated only in a small illumination area. Using the conventional OET manipulation method that relies solely on light induced DEP forces for manipulating, patterning, and transporting cells and microparticles across a large area is impractical. It has never been achieved in prior OET works. In SLOT, cell trapping and releasing functions are decoupled. A high-density array of phototransistors can be deployed over a large area for massively parallel trapping without being limited by the small FOV of manipulation optics. The optical projection system can serially scan across the entire SLOT platform, like a lithography stepper in modern semiconductor foundry, to pattern and manipulate single cells and microparticles across a large area.

Figure 2a presents the simulated electric field distribution and directions of DEP forces around a phototransistor with and without illumination respectively. At the dark phototransistors, DEP forces point toward their center electrodes, which is the underlying mechanism of self-locking. At the illuminated phototransistor, DEP forces point radially out of the center electrode to repel and release a trapped cell. The releasing direction is determined by the background flow. The landscape of potential energy profiles is shown in Fig. 2b. Without a background fluid flow, there could be regions where cells do not feel DEP forces (∇U ~ ∇E^2^ = 0, where U is the total potential energy for particle trapping). These include the centers of phototransistors where cells are locked, and the gaps between phototransistors, which are dead zones not optically tunable. Such dead zones can be minimized when phototransistors are positioned close enough. With an assisted background fluid flow providing constant viscous forces on particles, such dead zones can be completely removed. The introduction of a fluid viscous force shifts the trapping location from the phototransistor center to a new equilibrium point between the fluid force and the DEP force that point to opposite directions. When a light beam illuminates a phototransistor, the DEP force reverses its direction and points to the same direction as the background flow to release the microparticle to the downstream.

A unique feature of SLOT is its self-locking function over the entire active area in the dark state. The locking strength is determined by the dark-state voltage drop in the liquid layer, which is controlled by several parameters, including the thickness and dielectric constant of the insulation layer, AC frequency, and medium conductivity. A simple lump element model was constructed to analyze the optimal SLOT operation parameters in media of different conductivities (see Supplementary Fig. 1 for 9 different conditions). In an ideal SLOT operation, the dark-state voltage drop in the medium should be nearly 50% of the applied voltage. This is to ensure that sufficient DEP forces can be induced for cell locking in the dark state, while keeping enough room for voltage swing in the bright state to release cells. On a SLOT platform designed for manipulating cells and particles in high conductivity media, a 30 nm thick Al_2_O_3_ is chosen as the insulation layer for operation at a AC frequency range between 1 ~ 10 MHz.

**Fabrication of SLOT and Characterization of Phototransistor**. The fabrication of SLOT started from a boron-doped (1–10ohm-cm, 3 × 10^15^ cm^−3^) *p*-type single crystalline silicon substrate. A 1 μm SiO_2_ layer was grown on the substrate through thermal oxidation. The oxide layer was further patterned into an array of circular rings and, together with the remained photoresist, served as an ion implantation mask. Phosphorous ions were then introduced by a 2-step implantation process at 200/25 keV and with dose of 10^15^/4 × 10^15^ (Innovion Inc.) followed by rapid thermal annealing at 1100 °C for 120 seconds to form the resultant array of lateral ring-shaped NPN bipolar junction phototransistors. The projected implantation depth is 0.26 μm and the junction depth is 0.65 μm. Next, a 100 nm Au on 10 nm Ti metal stack is electron-beam evaporated onto the substrate surface followed by an oxide lift-off process to form electrodes self-aligned to and in contact with the underlying n^+^ regions. Finally, a 30 nm Al_2_O_3_ thin film was deposited via atomic layer deposition and subsequently patterned to create circular openings at the center electrodes of phototransistors for contact with aqueous media (see Supplementary Fig. 2 for detailed fabrication process flow).

To characterize the performance of the ring-shaped NPN phototransistors on SLOT, rectangular-shaped phototransistors were fabricated on the same wafers as the ring-shaped phototransistors for ease of measurement and characterization. An example of such testing structures was shown in the inserted figure in Fig. 3. Phototransistors of three different base widths (4 μm, 6 μm, and 8 μm) were tested. For the photocurrent measurement, optical illumination at a wavelength of 532 nm and an intensity of 1 W/cm^2^ is applied to all cases. Under a DC bias of 5 V, orders of magnitude higher photocurrents than the dark currents were observed for all cases. For phototransistors with a width less than 3 μm, significant dark leakage current was observed. For SLOT operation, a base width of 3.5 μm was chosen to provide high photocurrent yet tolerable dark current during operation. Phototransistors with different doping concentrations, implantation energy, and base width may have better performance in photosensitivity and dark leakage currents than the current design. However, such optimization process requires extensive parametric studies in simulation, device fabrication, and measurement, which are not included in the current work.

**Manipulation of microparticles and cells on SLOT**. To demonstrate SLOT’s capability for trapping a large number of microparticles and cells across an ultra-large area, a chip with a 1 cm^2^ active area was fabricated. The pitch between adjacent phototransistors was experimentally optimized to be 23 μm for trapping single mammalian cells of sizes between 8~15 μm. A shorter phototransistor pitch can result in interferences of electric fields between neighboring traps. Trapping experiments were carried out in an aqueous medium with a conductivity of 0.1 S/m. Before an AC voltage was applied, 8 μm microparticles were randomly distributed (Fig. 4a). When an AC voltage was turned on, without light illumination, all particles were self-locked into their neighboring phototransistors in less than 0.5 second over the entire 1 cm^2^ working area as shown in the zoom-in microscopy images in Fig. 4b. The trap fill up rate is ~40%, and a total of approximately 100,000 particles were trapped across this 1 cm^2^ platform with more than 250,000 phototransistors. Arranging randomly distributed microparticles into a periodic grid greatly facilitates later particle location addressing and optical releasing processes since all particles are at digital locations on the grid, and not anywhere in between. This result also proves that the dead zones between phototransistors where there are no DEP forces can be greatly minimized by having a high-density phototransistor array, even without the assistance of an external flow.

To demonstrate the selective releasing functions of SLOT, a biocompatible, double-side tape microfluidic channel was printed (Silhouette Portrait®, Silhouette America, Inc.) and sandwiched between an ITO substrate and a SLOT chip to introduce an external fluid flow. The tape-defined microfluidic channel is 400 μm in width, 60 μm in height, and 1 cm in length. The particle flow speed is controlled to be about 50 μm/s by an external syringe pump (PHD 2000, Harvard Apparatus, Inc.). As shown in Fig. 4c–f, a light beam scans across the SLOT chip to selectively release particles at 4 different regions to form letters “U”, “C”, “L” and “A”, demonstrating SLOT’s stepper mode operation. Released particles may be re-trapped in the downstream by empty phototransistors. The travelling distance of a released particle can vary from tens of micrometers up to millimeters before being re-trapped on the chip depending on the flow speed and the fill up rates in the downstream.

Single-cell manipulation in regular physiological buffer is critical for various biomedical applications. SLOT was designed to provide single-cell manipulation in high conductivity media, including regular physiological buffers, over a large area. In Fig. 5, single-cell manipulation under a fluorescence microscope was demonstrated on a SLOT platform with cells suspended in a regular cell culture buffer, DMEM media, with a conductivity of 1 S/m. The background flow was slowed down to roughly 20 μm/s such that a light beam can push a cell step-by-step to the downstream. Figure 5a shows how such a process was carried out to selectively manipulate a cell. A target cell and a non-target cell (Ramos Cells) were locked by two phototransistors on the platform. A laser beam, controlled by a 2D scanning mirror, was projected to release the target cell while keeping the non-target cell locked. The illuminated cell was released from its original trap, carried by the fluid flow to the downstream, and locked again by the next phototransistor. Figure 5b shows a 3 × 5 cell array formed by selectively and sequentially pushing single cells to target locations.

Cell viability and multi-day divisibility tests were also conducted. GFP HeLa cells were introduced onto the SLOT platform through a double-side tape channel. These cells were continuously trapped for 5 min., 10 min. and 20 min. before a fluorescence Propidium Iodide (PI) dye was introduced to check their viability on the chip. The results showed that cells in all three cases retain high viability (Fig. 6a). After each experiment, cells were released from the chip, collected on cell culture dishes, and transferred to an incubator for culturing to check their divisibility after trapping. Multi-day cell divisibility results show that the dividing rates of cells for all cases are similar to cells without SLOT treatment (Fig. 6b). These results show that cell trapping and manipulation on the SLOT platform over a short period of time does not have significant impacts on cell viability and divisibility. Long-term cell culturing on SLOT is currently not available since no appropriate culture media and gases exchange functions are integrated.

## Discussion

The demonstrated SLOT manipulation mechanism can be scaled up for single-cell manipulation across a much larger area and higher throughput than what has been demonstrated in the manuscript. The decoupled trapping and releasing function and the stepper mode operation are two key features that support such possibility. The manipulation area is limited by the size of wafers that can be manufactured. Since the fabrication of SLOT chips requires only two photolithography steps and micron scale feature sizes, they can be easily reproduced in an academic lab or mass-produced in any entry-level semiconductor manufacture foundry for low cost. If SLOT is fabricated on a 6” wafer, the active SLOT trapping area can be 500 cm^2^ and provide more than 100 million single-cell traps on a single wafer, which is estimated based on phototransistor density shown in the current manuscript.

A potential issue for such scaling-up operation is heat generation that will need to be managed by a cooling module underneath a SLOT chip. The power consumption on SLOT is dominated by the joule heating in the liquid layer, not the SLOT chip. The electrical power required to simultaneously activate 250,000 traps on a 1 cm^2^ SLOT platform is about 0.6 W in a medium with a conductivity of 0.1 S/m, or 6 W in regular physiological buffers. The power consumption scales up linearly with the number of parallel operating traps and the medium conductivity. Single crystalline silicon has an excellent heat transfer property. It has a thermal conductivity of 149 W/m·K, which is 2 orders of magnitude higher than glass (1 W/m·K) and amorphous silicon (0.5 W/m·K) that are used in conventional OET. Assuming the tolerable temperature rise at the SLOT surface in contact with liquid is 2.5 °C, and the silicon substrate thickness is 0.5 mm, the heat dissipation rate is 74.5 W/cm^2^, which is more than 10 times larger than the 6 W/cm^2^ heat generation rate for SLOT operation in regular physiological buffer. This also means the approximate temperature rise in the current SLOT chip is less than ~0.25 °C for media of electrical conductivity less than 1 S/m. Potential applications of SLOT are broad, including tissue engineering^28^,^29^,^30^, drug screening^31^, cell-to-cell communication, rare cell sorting, *in vitro* fertilization^32^ and beyond.

## Methods

### Laser Scanning System

A 532 nm, 10 mW green laser (Crystalaser Inc.) was used as the illumination light source to trigger phototransistors on SLOT. A two-axis scanning mirror system (Cambridge Technology Inc.) controlled by LabVIEW was utilized to generate scanning light patterns.

### Optical System

SLOT utilized non-transparent silicon as the substrate. A 20× objective lens with a 3 cm long working distance was used for optical observation. A dichromic mirror is placed between the objective lens and the SLOT device to reflect the laser beam for triggering phototransistors.

### Finite-Element Analysis

The 2D numerical simulation results are generated from COMSOL Multiphysics. The conductivity and relative dielectric constant of silicon are set to be 10^−5^ S/m and 4.5, respectively. To represent the photoconductivity at the illuminated regions, we varied the conductivity of p-type silicon from 10^−5^ to 10^−1^ S/m. Aqueous liquid layer was assumed to have a conductivity of 1 S/m and a dielectric constant of 80. The Al_2_O_3_ insulation layer has a conductivity of 10^−14^ S/m and a dielectric constant of 12. The overall electrical potential is set to be 10 Vpp and 10 MHz.

## Additional Information

**How to cite this article**: Yang, Y. *et al.* Self-Locking Optoelectronic Tweezers for Single-Cell and Microparticle Manipulation across a Large Area in High Conductivity Media. *Sci. Rep.* **6**, 22630; doi: 10.1038/srep22630 (2016).

## Supplementary Material

Supplementary Information

Supplementary Video 1

Supplementary Video 2

## Figures and Tables

**Figure 1:**
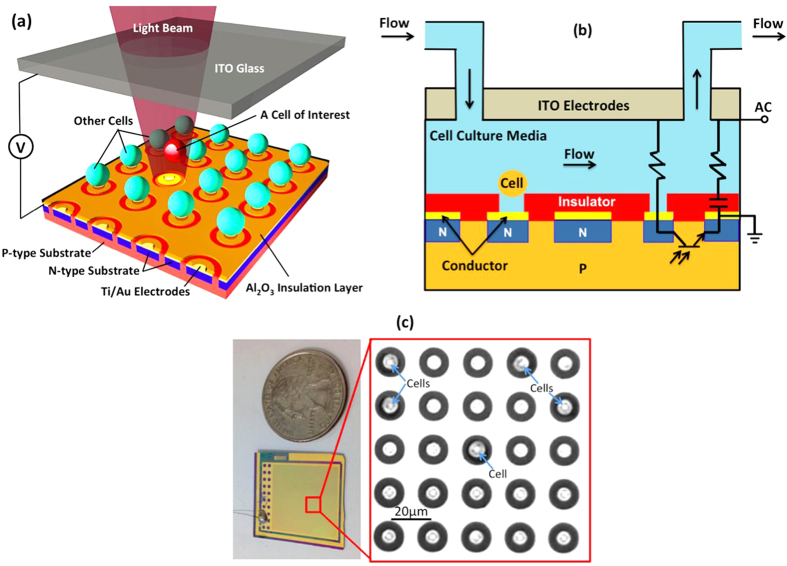
Schematic and Prototype of a Self-Locking Optoelectronic Tweezers (SLOT) Platform. (**a**) SLOT utilizes an array of ring-shaped phototransistors as optical sensors to control the respective DEP traps. A 30 nm thick Al_2_O_3_ dielectric layer is coated on the SLOT surface to ensure partial voltage drop in the dark state to realize the single-cell self-locking function in high conductivity media. (**b**) AC voltage is applied between the top ITO electrode and the bottom conductor layer (Not the p-type substrate). Electrical currents flow laterally through NPN bipolar transistors and partially through the Al_2_O_3_ insulator into the liquid layer. Optical illumination on a phototransistor turns off its DEP locking function and releases the trapped cell via background fluid flow. (**c**) A photo of a fabricated SLOT chip and a snapshot microscopy image (taken from supplementary video 1) shows the ring-shape phototransistors, center floating electrodes, and single cells trapped on it.

**Figure 2:**
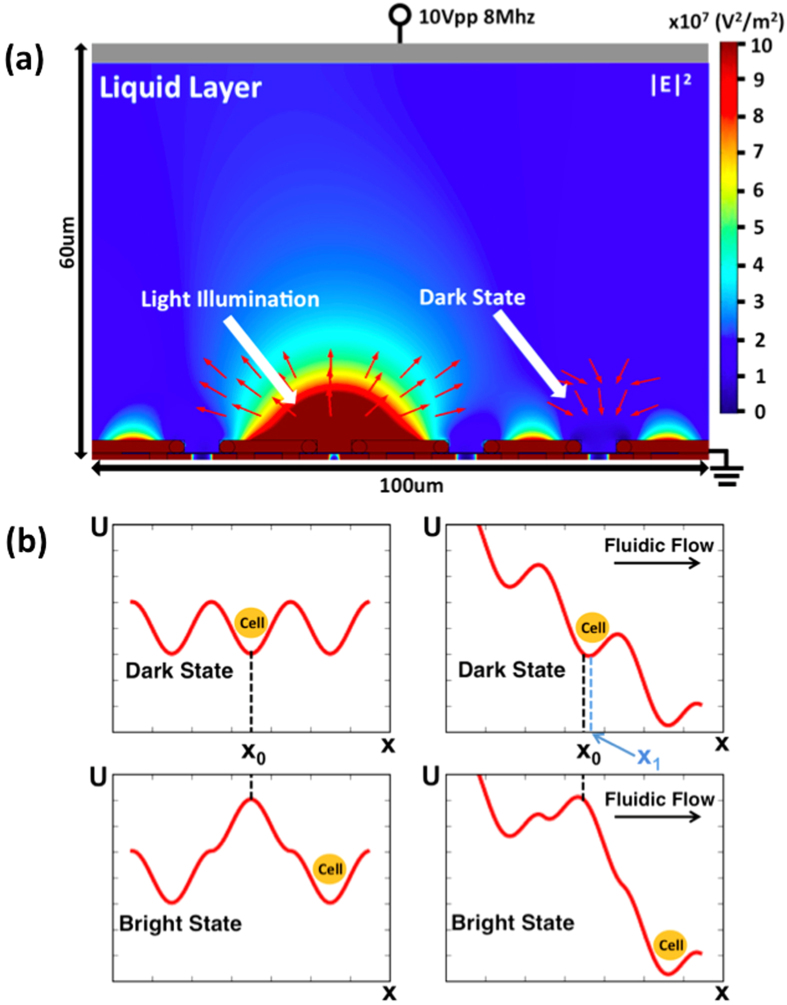
SLOT Operating Modeling. (**a**) Numerical simulation of DEP forces around phototransistors. SLOT operation at an appropriate AC frequency ensures partial voltage drop in the medium without light illumination. At phototransistors in the dark state, DEP forces point toward the centers of phototransistors, displaying the underlying principle of self-locking. At the illuminated phototransistor, the direction of DEP forces reverses and repels the trapped particle away. (**b**) Since the phototransistor has a radial symmetric shape, the induced DEP forces are also symmetric. Without an external flow, there are regions where there are no potential gradients and zero DEP forces, including the centers of the phototransistors, location x_o_, and gaps between phototransistors. With a background flow, the new trapping location offsets to a new balanced point x_1_. Under illumination, the DEP force direction reverses to release the cell that is carried to the downstream by the background flow.

**Figure 3:**
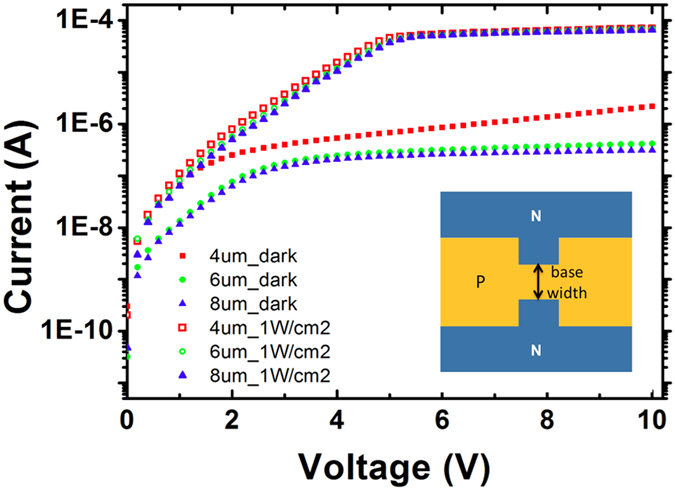
SLOT I-V Curve Measurement. Current-voltage characteristics are measured based on a rectangular-shaped phototransistor test structure. NPN phototransistors of three different base widths, 4 μm, 6 μm, and 8 μm, were tested. Photocurrent is measured under the illumination of a 532 nm green laser beam at an intensity of 1 W/cm^2^. The phototransistor with a 4 μm base width gives a higher photocurrent than others, but comes with a higher dark current too. In SLOT devices, base widths between 3.5 μm to 4 μm are typically used. For base width shorter than 3 μm, dark current becomes too high and affects the self-locking function.

**Figure 4:**
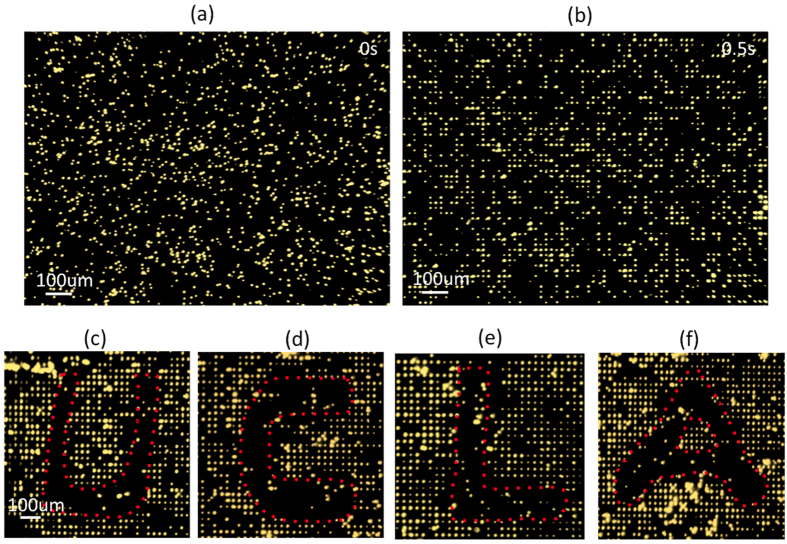
Large-Area Parallel Trapping and Optical Releasing (“Stepper” Mode). (**a–b**) Parallel trapping of 8 μm polystyrene beads on a 1 cm^2^ SLOT platform with more than 250,000 phototransistor traps. The experiment is conducted in a sodium chloride medium with a conductivity of 0.1 S/m. The applied AC voltage is 5 V_pp_ at a frequency of 500 kHz. Randomly distributed microparticles are all self-locked into nearby traps within 0.5 second after an external voltage was turned on. The trap fill rate is about 40% and over 100,000 particles are simultaneously trapped on this platform. (**c–f**) A biocompatible double-side tape microfluidic channel was sandwiched between an ITO glass and a SLOT chip to provide a background fluid flow. A scanning light beam selectively releases part of the trapped particles in four different regions on the chip to form the four letters “U”, “C”, “L”, and “A”.

**Figure 5:**
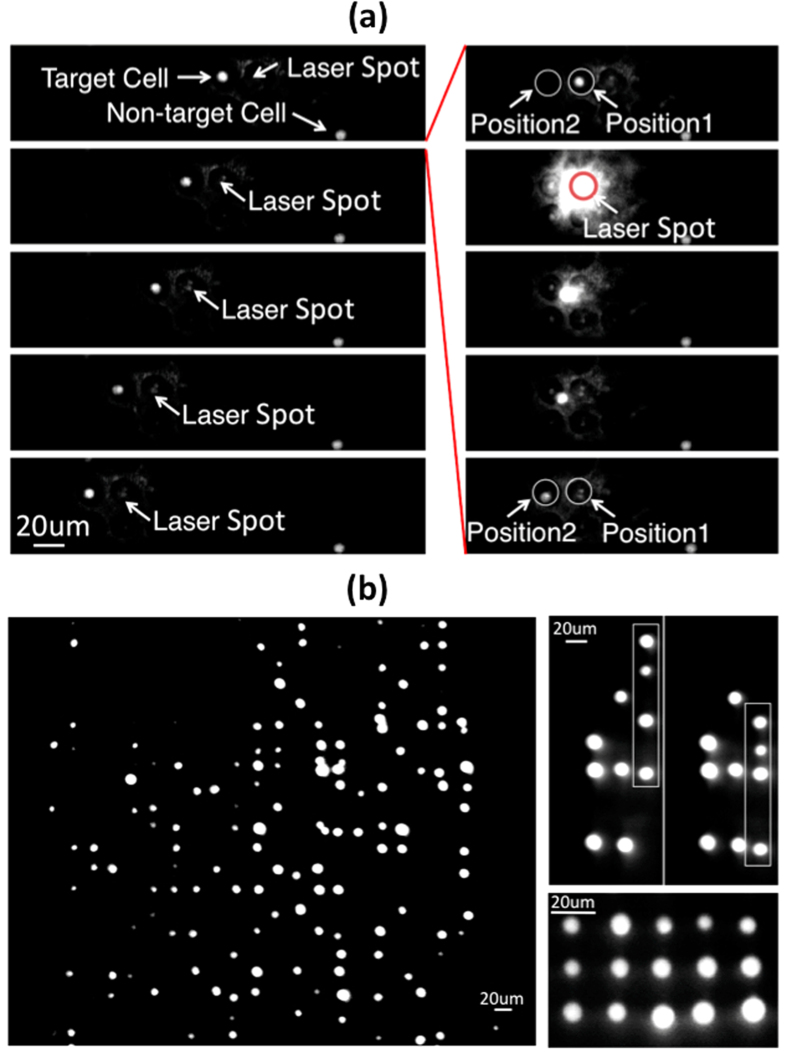
Single Cell Manipulation in a Regular Cell Culture Medium. (**a**) Left: stepwise releasing of a trapped target single cell to the downstream phototransistor traps. Right: detailed process showing how a target cell was released from Position 1 and trapped by the next phototransistor in the downstream (Position 2) (See Supplementary Video 2). This experiment is conducted in DMEM (1 S/m) under 10 V_pp_ AC voltage at 8 MHz. (**b**) Such a stepwise function allows the assembly of single cells into a desired pattern such as the shown 3 × 5 array on a SLOT platform.

**Figure 6:**
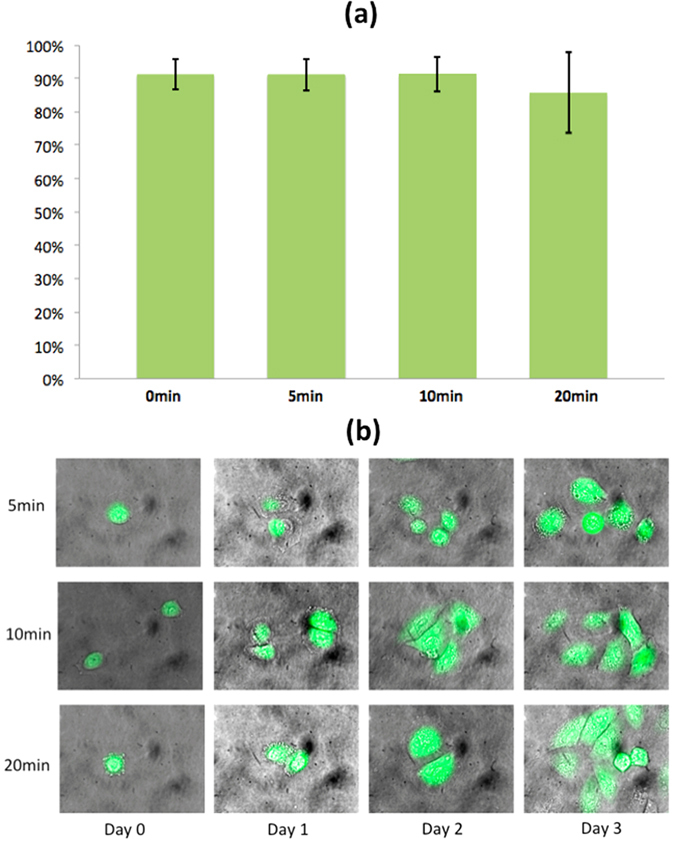
Cell Viability and Cell Divisibility. (**a**) Three different batches of GFP HeLa cells were introduced onto a SLOT platform and continuously trapped for 5 min., 10 min. and 20 min. in Dulbecco’s Modified Eagle’s Medium (DMEM), respectively, under an AC bias of 10 V_pp_ at 8 MHz. PI dye was then introduced to stain the dead cells. Cells from all three experiments retained high cell viability ( > 85%). (**b**) Cells were further transferred to an incubator for a 3-day divisibility analysis. Cells from all three experiments divided at a normal rate (24hrs~36hrs).
