# Supplementary material for: Self-Locking Optoelectronic Tweezers for Single-Cell and Microparticle Manipulation across a Large Area in High Conductivity Media
Source: Sci Rep. 2016 Mar 4;6:22630. doi: 10.1038/srep22630 (PMC4778053; doi:10.1038/srep22630)
Supplement: Supplementary Information [file srep22630-s1.doc]

Self-Locking Optoelectronic Tweezers for Single-Cell and Microparticle Manipulation across a Large Area in High Conductivity Media

Yajia Yang^a^, Yufei Mao^b^, Kyeong-Sik Shin^b^, Chi On Chui^b,c^ and Pei-Yu Chiou^a,c*^

^a^ Mechanical and Aerospace Engineering Department, University of California, Los Angeles, USA

^b^ Electrical Engineering Department, University of California, Los Angeles, USA

^c^ Bioengineering Department, University of California, Los Angeles, USA

**Supplementary Materials**


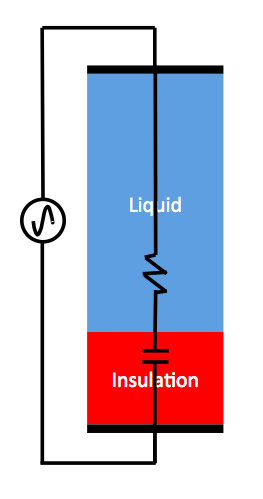

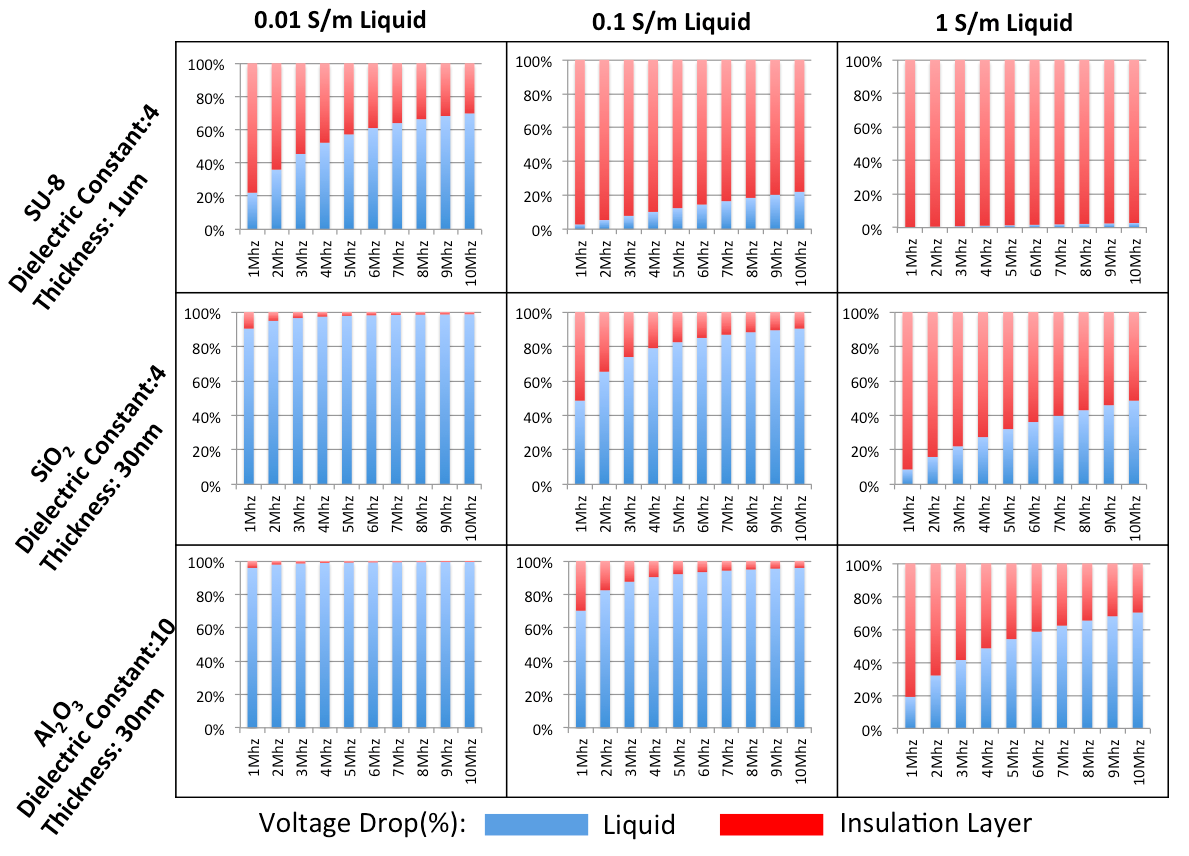


**Fig. 1 Comparison of the Effects of Different Insulation Layers in SLOT operation.** The partial leak voltage drops are calculated for 9 different combinations of insulation layers and liquid conductivities. Based on these calculations we identify that 30 nm Al_2_O_3_ (ε=12) should be ideal for SLOT operation in 1 S/m high conductive media.


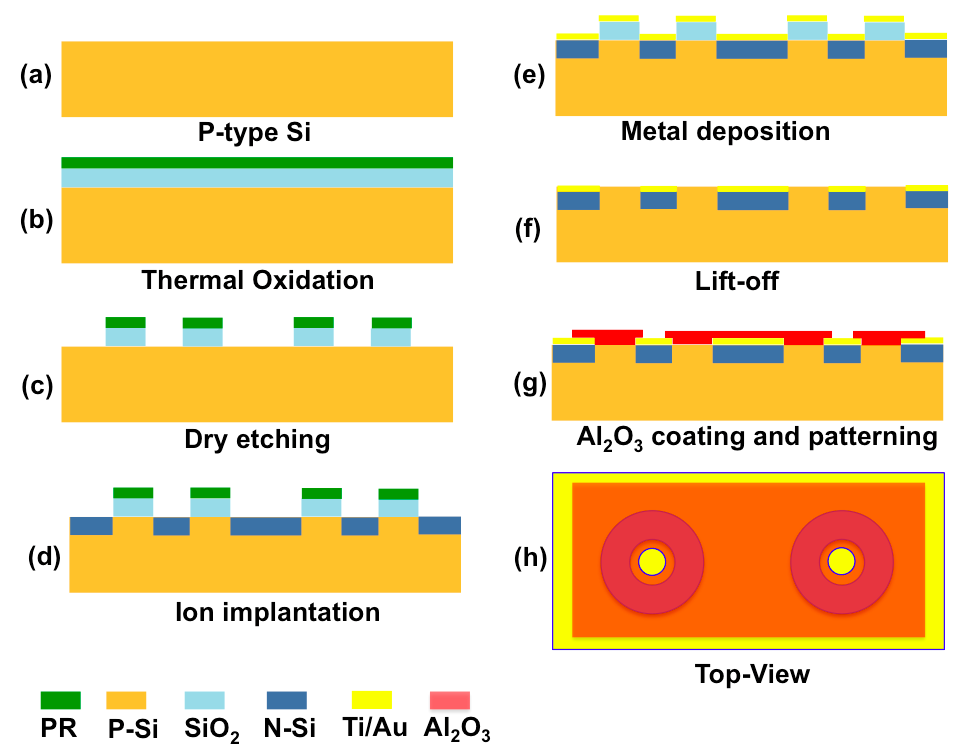


**Fig. 2. Fabrication Process Flow of SLOT.** (a) The process starts with a boron-doped (1-10ohm-cm, 3x10^15^cm^-3^) p-type single crystalline silicon substrate. (b) 1 μm SiO_2_ is grown above the substrate through thermal oxidation. (c) The silicon dioxide and the photoresist layers are patterned to form a ring-shaped implantation mask through dry etching. (Mask 1) (d) Phosphorous ions were then introduced by a 2-step implantation process at 200/25keV and with dose of 10^15^/4×10^15^, followed by rapid thermal annealing at 1100 °C for 120 seconds to form lateral ring-shaped NPN bipolar junction phototransistors. (e) A 100 nm/10 nm Au/Ti layer is evaporated. (f) The silicon dioxide islands were removed to leave behind Au/Ti electrodes above the phototransistors’ n^+^ regions. (g) An Al_2_O_3_ insulation layer is deposited with the atomic layer deposition (ALD) approach and patterned to create a circular opening in the center of each phototransistor. (Mask 2) (h) Top-view of fabricated phototransistors on a SLOT device.

**Caption of Supplementary Videos**

Supplementary video 1: Self-locking of single cells into phototransistor traps. GFP HeLa cells were self-locked into phototransistor traps on SLOT in Dulbecco’s Modified Eagle’s Medium (DMEM) under an AC bias of 10 Vpp at 8 MHz.

Supplementary video 2: Stepwise releasing of a trapped target single cell to the downstream traps.
